# Diversity of an Odonata assemblage from a tropical dry forest in San Buenaventura, Jalisco, Mexico (Insecta, Odonata)

**DOI:** 10.3897/BDJ.12.e116135

**Published:** 2024-02-23

**Authors:** Enrique González Soriano, Felipe Noguera, Cisteil X Pérez-Hernández

**Affiliations:** 1 Departamento de Zoología, Instituto de Biología, Universidad Nacional Autónoma de México, Mexico City, Mexico Departamento de Zoología, Instituto de Biología, Universidad Nacional Autónoma de México Mexico City Mexico; 2 Estación de Biología Chamela, Instituto de Biología, Universidad Nacional Autónoma de México, San Patricio, Jalisco, Mexico Estación de Biología Chamela, Instituto de Biología, Universidad Nacional Autónoma de México San Patricio, Jalisco Mexico; 3 Laboratorio de Ecología de la Conducta, Facultad de Biología, Universidad Michoacana de San Nicolás de Hidalgo, Morelia, Mexico Laboratorio de Ecología de la Conducta, Facultad de Biología, Universidad Michoacana de San Nicolás de Hidalgo Morelia Mexico

**Keywords:** richness, temporal diversity, phylogenetic diversity, abundance, Odonata assemblage, tropical dry forest

## Abstract

**Background:**

The patterns of richness, diversity, and abundance of an odonate assemblage from San Buenaventura, Jalisco are presented here. A total of 1087 specimens from seven families, 35 genera and 66 species were obtained through monthly samplings of five days each during a period of one year. Libellulidae was the most diverse family (28 species), followed by Coenagrionidae (21), Gomphidae (7), Aeshnidae (6), Calopterygidae (2), Lestidae (1) and Platystictidae (1). *Argia* was the most speciose genus. The highest species richness and Shannon diversity were found during August and September, whereas the highest abundance was observed in June and the highest Simpson diversity was recorded in September — all of which were associated with the rainy season. The highest values of phylogenetic diversity were found from June to October. The different diversity facets of this assemblage were positively correlated with precipitation and minimum temperature, whereas maximum temperature showed no influence. In addition, we found that this odonate diversity was higher than most Mexican localities with tropical dry forest (TDF) studied.

**New information:**

We continue our efforts to describe the patterns of richness, diversity and abundance of some insect groups associated with the tropical dry forest ecosystem in Mexico, following a latitudinal gradient of the distribution of this ecosystem in the country. Our emphasis here was to evaluate the spatial and temporal patterns of richness and diversity of an Odonata assemblage from Jalisco, Mexico.

## Introduction

This study continues our efforts to describe the patterns of richness, diversity and abundance of some insect groups associated to the tropical dry forest in Mexico (e.g.Noguera et al. 2002, Zaragoza–Caballero et al. 2003, Noguera et al. 2007, González-Soriano et al. 2008, González-Soriano et al. 2009, González-Soriano et al. 2021). Tropical dry forests (abbreviated hereinafter as TDFs) are defined as forests with pronounced seasonality in rainfall distribution, resulting in several months of drought (Mooney et al. 1995). TDFs are highly diverse ecosystems that harbour a large number of endemic species not found in any other ecosystem in the world. These forests also face an intense pressure due to deforestation and the conversion of original lands into lands for agriculture and cattle raising, specially in Latin America (Sánchez‐Azofeifa et al. 2005, DRYFLOR et al. 2016). In Mexico, the extension of TDFs has been dramatically diminishing over several years ago at an amazing rate (Miles et al. 2006). To protect and conserve these rich ecosystems is an urgent issue, by means of the study of their biodiversity before it disappears. For this and other reasons, we — a group of entomologists from the Institute of Biology at the National Autonomous University of Mexico — started a project since 1997, with the aim of identifying the spatial and temporal patterns of richness and diversity of selected groups of insects through a latitudinal gradient of the distribution of this ecosystem in Mexico. Previous studies have been performed at several sites along the Pacific Mexican Coast and at some sites at south Central Mexico (e.g. Noguera et al. 2002, Zaragoza–Caballero et al. 2003, Noguera et al. 2007, González-Soriano et al. 2008, González-Soriano et al. 2009, Noguera et al. 2009, Noguera Martínez et al. 2012, Noguera et al. 2017). Here, we present the results of a faunistic study made during 1996-1997 at one of these sites: San Buenaventura, Jalisco, in central-western Mexico.

## Materials and methods

### Study site

**Figure 1.**
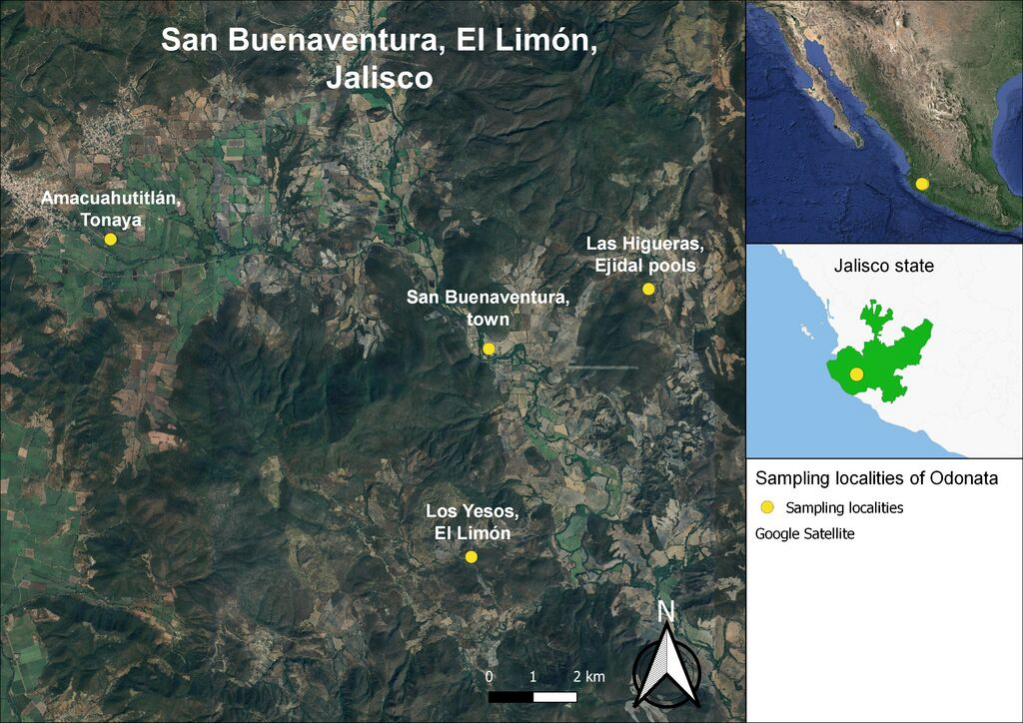
Sampling localities of Odonata species in San Buenaventura, Jalisco, Mexico. Entomological samplings were performed from 1996-1997. Imagery 2015, INEGI Maxar Technologies CNES/Airbus. Downloaded August 2023.

**Figure 2.**
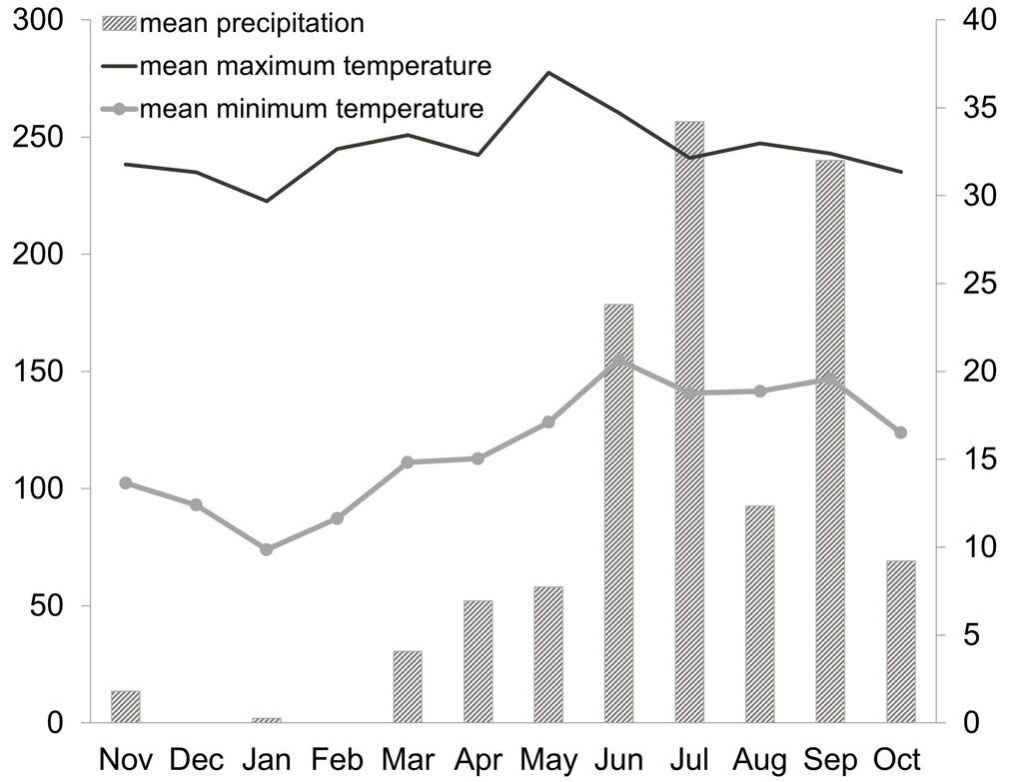
Monthly variation of rainfall and temperature in San Buenaventura, Jalisco during 1996-1997.

San Buenaventura (from here on SBV) is located on the eastern slope of the Sierra de Cacoma-Sierra de Manantlan, Jalisco, Mexico (latitude 19°45'19'', 19°48'50'' N and longitude -104°01'25'', -104°08'25'' W; Fig. 1). The climate is warm sub-humid type according to the Köeppen climate classification modified by García (1988). The average annual precipitation from the nearest weather station Presa Basilio Vadillo (Comisión Nacional del Agua and Servicio Metereológico Nacional 2023) was 747 mm, while the average air temperature was 23.8°C, with average maximum and minimum temperatures of 31.9°C and 15.8°C respectively. The highest temperature during the study period was recorded in May and the lowest in January (Fig. 2). The dominant vegetation in the area is TDF. The dominant tree species are *Lysilomaacapulcense* (Kunth) Benth (Fabaceae), *L.divaricatum* (Jacq.) J.F.Macbr, *Jacaratiamexicana* A. DC. (Caricaceae), *Amphipterygiumadstringens* (Schltdl.) Standl. (Anacardiaceae), *Entadapolystachya* (L.) DC. (Fabaceae), *Ceibaaesculifolia* (Kunth) Britten & Baker f. (Malvaceae), *Senegaliamacilenta* (Rose) Britton & Rose (Fabaceae), *Vitexmollis* Kunth (Lamiaceae), *Ipomoeabracteata* Cav. (Convolvulaceae), *Bursera* spp. (Burseraceae) and *Cochlospermumvitifolium* (Willd.) Spreng. (Bixaceae) (Jardel 1992). A gallery forest, characterised by trees taller than those of the TDF, extends along streams and narrow canyons. Flat areas in the zone have been open to agriculture and hillsides are used as grazing areas for cows and goats, which has resulted in the near disappearance of the native understorey.

### Sampling methods and regimes

The study area is located within the Ayuquila-Armeria River Basin and, more specifically within the Tuxcacuesco River and Ayuquila sub-basins (Guevara Gutiérrez et al. 2019, Rodríguez-Contreras et al. 2019). Sampling collections were done around three main localities: SBV town, Los Yesos and Amacuahutitlán, and only occasionally at Las Higueras (see Table 1, Fig. 1).

**Figure 3.**
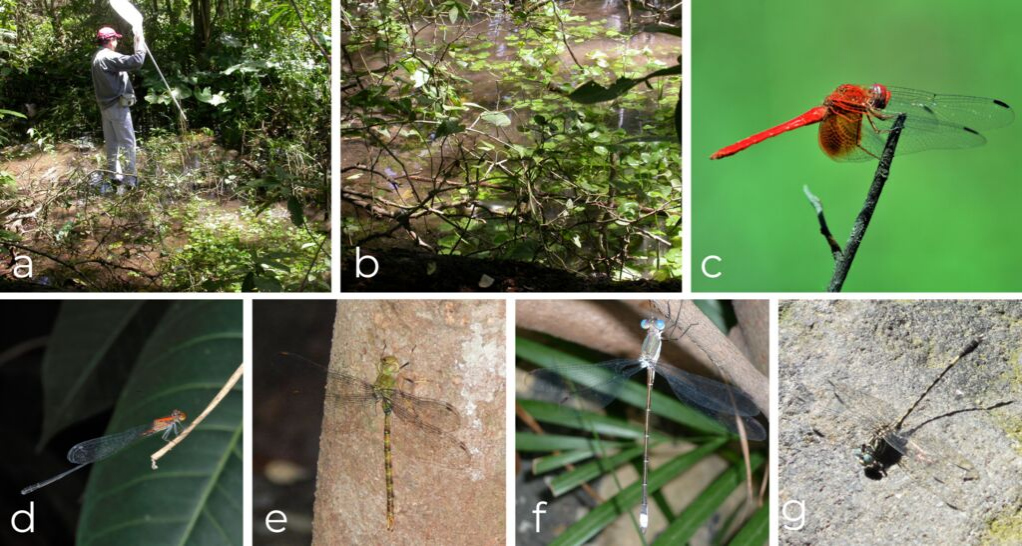
Habitats and Odonata species from four localities in San Buenaventura, Jalisco, Mexico; **a, b** microhabitat of *Anisagrionallopterum*; **c** *Dythemismaya*; **d** *Neoneuraamelia*; **e** *Gynacanthahelenga*; **f** *Archilestesgrandis*; **g** *Progomphusclendoni*. Photos: a, b, César Durán; c, f, g Enrique González Soriano; d, Enrique Ramírez; e, Eric Hough (Naturalista).

The SBV samplings were made along the margins of the Ferreria River, a permanent river that crosses the town. Samplings in Amacuahutitlán and Los Yesos were done at small open streams and finally, at the site of Las Higueras consisting of a small, mostly shaded, spring-fed shallow stream with abundant aquatic plants on its surface (Fig. 3). Most collecting sites belong to the Municipality of El Limón, except for Amacuahutitlán, which is located within the Municipality of Tonaya (see Table 1).

Fieldwork in SBV was always conducted by two people between November 1996 and October 1997. Collections were carried out for a period of five days every month. Specimens were obtained through direct collecting, between 09:00 h and 15:00 h (10:00 h-16:00 h in the daylight-saving time).

All the Odonata records collected in SBV, Jalisco, Mexico for this work were included in the GBIF dataset Digitization and Systematization of the National Biological Collections of the Institute of Biology, UNAM from the National Autonomous University of Mexico (Sánchez Cordero Dávila 2023). Data on the phenology and the number of specimens collected (between parentheses) were added in the Odonata species list (Results section); additional information can be consulted in the Supplementary materials and photographs of some species in Fig. 3.

### Diversity analysis

The diversity of the SBV odonate assemblage was analysed through different metrics of species and phylogenetic diversities. We first quantified:

(a) abundance, measured as the number of specimens collected through a species rank abundance curve;

(b) species richness, as the number of species observed (diversity order 0, ^0^D);

(c) Shannon diversity, which corresponds to the exponential of the Shannon Index (diversity order 1, ^1^D); and

(d) Simpson diversity, which corresponds to the inverse of the Simpson Index (diversity order 2, ^2^D) (Jost 2006, Chao et al. 2014).

The measurement unit for ^1^D and ^2^D is the number of effective species, also referred to as Hill numbers, in such a way that ^1^D indicates the effective number of equally abundant species within an assemblage and ^2^D showed the effective number of the most abundant or most dominant equally abundant species.

We then calculated the maximum expected richness value of diversity for ^0^D, ^1^D and ^2^D to compare those values with our observed sample. We used the Spade R package (Chao et al. 2015) to compute the non-parametric abundance-based Chao 1-bias corrected estimator and the estimators proposed by Magurran for ^0^D, ^1^D and ^2^D, respectively (Magurran 1988, Chao 2005, Chao et al. 2013). We also calculated a cumulative species curve for the whole Odonata assemblage from SBV, through the interpolation-extrapolation method proposed by Chao et al. (2014) for ^0^D, ^1^D and ^2^D and using the iNEXT R package (Hsieh et al. 2016).

In addition, we evaluated monthly abundance, ^0^D, ^1^D, and ^2^D to analyse temporal diversity patterns in the Odonata assemblage. In addition, we analysed temporal phylogenetic diversity through the taxonomic diversity (Δ) and taxonomic distinctness (Δ*) indices, which are based on the abundance and the average taxonomic distinctness (Δ+) index, based on species incidence (Warwick and Clarke 1995, Clarke and Warwick 2001). Taxonomic diversity and taxonomic distinctness analyse phylogenetic divergence amongst species within communities or assemblages according to their topological organisation, i.e. the phylogenetic relationships amongst taxa and their taxonomic hierarchy; these measures calculate how closely related the specimens (Δ) or the species within the assemblage are (Δ*) or how evenly distributed their evolutionary paths are through the taxonomic hierarchy (Δ+) (Clarke and Warwick 1998, Clarke and Warwick 2001). Since the most recent odonate phylogenies do not include all taxa within the order (e.g. Bybee et al. (2021)), we used the hierarchical classification above species-level as a proxy to calculate taxonomic distances amongst taxa using diferent phylogenetic diversity metrics (Clarke and Warwick 2001; Tucker et al. 2016). In particular, the phylogenetic hypothesis of Bybee et al. (2021) for suprafamily levels and Dijkstra et al. (2014) and Carle et al. (2015) for suprageneric levels of Anisoptera and Zygoptera suborders, respectively were used for this purpose. We included seven taxonomic levels to evaluate monthly phylogenetic divergence within the SBV odonate assemblage: order, suborder, superfamily, subfamily, tribe, genus and species. Phylogenetic divergence was calculated through the *taxondive* and *taxa2dist* functions of the Vegan R package (Oksanen et al. 2015).

### Relationship between odonate diversity and abiotic factors

To analyse whether species and phylogenetic diversity of the SBV odonate assemblage are related to abiotic factors, we performed Pearson’s correlation analyses between monthly species diversity (abundance, ^0^D, ^1^D and ^2^D), monthly phylogenetic diversity (Δ, Δ*, Δ+) and monthly mean rainfall and temperature documented in SBV, Jalisco during the sampling time. Values of precipitation and temperature were obtained from the closest weather station (Presa Basilio Vadillo) through the National Meteorological System (Comisión Nacional del Agua and Servicio Metereológico Nacional 2023). We found a positive correlation between temperature and precipitation. Pearson’s correlation analyses were done in Past software (Hammer et al. 2001). In addition, we generated a heatmap using the *pheatmap* R package (Warnes et al. 2012) to display the presence and abundance that each odonate species showed monthly. Monthly diversity and phylogenetic divergence analyses allowed us to evaluate how the species diversity and the taxonomic assemblage structure were related to monthly changes of temperature and humidity. In other words, those analyses allowed us to evaluate how the abiotic factors can be associated with the temporal structure of the Odonata community of the TDF.

## Checklists

### List of Odonata species registered from San Buenaventura, Jalisco, Mexico

#### Archilestes grandis

(Rambur, 1842)

297139C6-D11D-503C-B9F1-7EAA5319E5E3

##### Distribution

San Buenaventura, Amacuahutitlan, Jalisco, MX

##### Notes

Phenology in SBV: Aug (2), Sep (3), Oct (4).

#### Palaemnema domina

Calvert, 1903

93BED9E6-2A5A-538F-BD90-84CF7F27A024

##### Distribution

Las Higueras, Los Yesos, Jalisco, MX

##### Notes

Phenology in SBV: Sep (2).

#### Hetaerina americana

(Fabricius, 1798)

3BFA7694-EB48-5161-A2CB-05D42B5D4C92

##### Distribution

San Buenaventura, Amacuahutitlan, Las Higueras, Jalisco, MX

##### Notes

Phenology in SBV: Nov (2), Dec (18), Jan (7), Feb (13), Mar (22), Apr (4), May (2), Jun (61), Jul (1), Aug (5), Sept (6), Oct (8).

#### Hetaerina capitalis

Sélys 1873

744C2893-22E4-5FD5-A4FB-E0CC17A510BF

##### Distribution

San Buenaventura, Jalisco, MX

##### Notes

Phenology in SBV: Jul (1)

#### Anisagrion allopterum

Sélys, 1876

6A3140CE-D21A-579E-BBFB-DCD3FB618512

##### Distribution

Las Higueras, Jalisco, MX

##### Notes

Phenology in SBV: Aug (3), Sept (1)

#### Apanisagrion lais

(Brauer in Sélys, 1876)

33552AA9-2953-530E-AF8B-0486612CB940

##### Distribution

San Buenaventura, Amacuahutitlan, Jalisco, MX

##### Notes

Phenology in SBV: Jan (3), Mar (1), Apr (2), Jul (1), Sept (1), Oct (1)

#### Argia anceps

Garrison, 1996

FE547AB6-7714-5AC4-9514-AFB3784DD109

##### Distribution

San Buenaventura, Amacuahutitlan, Las Higueras, Los Yesos, Jalisco, MX

##### Notes

Phenology in SBV: Dec (3), Jan (1), Feb (1), Apr (1), May (3), Jun (8), Jul (4), Aug (6), Sept (10), Oct (1).

#### Argia carlcooki

Daigle, 1995

340E77B5-2E50-5453-881F-3FF9F7B7F6F7

##### Distribution

Amacuahutitlan, Las Higueras, Jalisco, MX

##### Notes

Phenology in SBV: Mar (3), Apr (1), Jun (1), Aug (4), Sept (1), Oct (1)

#### Argia extranea

(Hagen, 1861)

1E41F6E7-639A-5310-A5EA-B557C637F259

##### Distribution

San Buenaventura, Amacuahutitlan, Las Higueras, Jalisco, MX

##### Notes

Phenology in SBV: Feb (13), Mar (2), Apr (10), May (1), Jun (11), Aug (7), Sept (6), Oct (9)

#### Argia harknessi

Calvert, 1899

38C142BD-3B27-5A25-9016-6470739D1001

##### Distribution

San Buenaventura, Las Higueras, Los Yesos, Jalisco, MX

##### Notes

Phenology in SBV: Nov (1), Dec (3), Jan (6), Feb (10), Mar (7), Apr (2), Jun (7), Jul (1), Aug (1), Sep (5), Oct (1)

#### Argia mayi

González-Soriano, 2012

9F7F8CF3-E836-51A2-A450-26629BC4EDE1

##### Distribution

Las Higueras, Jalisco, MX

##### Notes

Phenology in SBV: Aug (5)

#### Argia oculata

Hagen in Sélys, 1865

EC0049E7-3F01-590F-8A07-64BBA8C19560

##### Distribution

San Buenaventura, Amacuahutitlan, Los Yesos, Jalisco, MX

##### Notes

Phenology in SBV: Feb (1), Mar (1), Apr (2), Jun (1), Aug (1), Sept (3)

#### Argia oenea

Hagen in Sélys, 1865

325C52DB-D7A9-5124-8504-0FD27EB229C8

##### Distribution

San Buenaventura, Amacuahutitlan, Los Yesos, Jalisco, MX

##### Notes

Phenology in SBV: Dec (2), Jan (1), Aug (1)

#### Argia pallens

Calvert, 1902

E6256684-8D89-5F21-A890-DDD3963CEDD2

##### Distribution

San Buenaventura, Amacuahutitlan, Jalisco, MX

##### Notes

Phenology in SBV: Mar (2), Apr (1), Jun (3), Sep (3)

#### Argia pulla

Hagen in Sélys, 1865

356C16A5-2068-52E0-A750-4CAB7065EA6A

##### Distribution

San Buenaventura, Amacuahutitlan, Las Higueras, Los Yesos, Jalisco, MX

##### Notes

Phenology in SBV: Dec (3), Jan (3), Feb (11), Mar (12), Apr (8), May (1), Jun (12), Aug (6), Sep (3), Oct (9)

#### Argia tezpi

Calvert, 1902

9CD6F0DB-5B78-5A2E-AC08-A6A65FA27D83

##### Distribution

San Buenaventura, Las Higueras, Los Yesos, Jalisco, MX

##### Notes

Phenology in SBV: Dec (3), Jan (3), Feb (5), Mar (5), May (1), Jun (5), Jul (2), Aug (1), Sep (3), Oct (1)

#### Enallagma civile

(Hagen, 1861)

116A8C2E-6A3A-5523-86AE-30745A9F62E8

##### Distribution

San Buenaventura, Los Yesos, Jalisco, MX

##### Notes

Phenology in SBV: Dec (2), Apr (2)

#### Enallagma novaehispaniae

Calvert, 1907

91008167-F49E-53AF-8587-300E1B19CDE9

##### Distribution

San Buenaventura, Jalisco, MX

##### Notes

Phenology in SBV: Nov (2), Dec (6), Jan (5), Feb (8), Mar (4), Apr (5), Jun (13), Aug (1), Sep (4), Oct (1)

#### Enallagma semicirculare

Sélys, 1876

971C83A5-DCEA-5921-B4AE-D53F25CDBB03

##### Distribution

San Buenaventura, Jalisco, MX

##### Notes

Phenology in SBV: Dec (2), Feb (3), Apr (6)

#### Ischnura hastata

(Say, 1840)

3C242BF1-96D0-5215-A45B-046BC0342818

##### Distribution

San Buenaventura, Amacuahutitlan, Jalisco, MX

##### Notes

Phenology in SBV: Dec (2), Feb (2), Mar (4), Apr (6), May (3), Jun (4).

#### Ischnura ramburii

(Sélys, 1850)

DE599C3F-E26E-5C38-B8E2-507A341E4AA8

##### Distribution

San Buenaventura, Amacuahutitlan, Jalisco, MX

##### Notes

Phenology in SBV: Mar (1), Apr (4)

#### Neoneura amelia

Calvert, 1903

28BBD4A6-6E16-5DF4-B249-D0A14B649964

##### Distribution

San Buenaventura, Amacuahutitlan, Jalisco, MX

##### Notes

Phenology in SBV: Dec (1), Jun (1), Jul (2), Sep (2), Oct (6)

#### Protoneura cara

Calvert, 1903

83F36F35-6BE5-5296-9D56-10C5681F1A89

##### Distribution

San Buenaventura, Amacuahutitlan, Jalisco, MX

##### Notes

Phenology in SBV: Nov (1), Dec (2), Feb (7), Mar (3), May (1), June (2), Jul (2), Sep (4), Oct (6)

#### Telebasis levis

Garrison, 2009

92621B43-DA6D-5783-B8B3-92A357DF5AEE

##### Distribution

San Buenaventura, Jalisco, MX

##### Notes

Phenology in SBV: Jun (7)

#### Telebasis salva

(Hagen, 1861)

6840812B-E2F3-5DB6-9909-E39C57599A1A

##### Distribution

San Buenaventura, Amacuahutitlan, Las Higueras, Jalisco, MX

##### Notes

Phenology in SBV: Nov (1), Dec (5), Jan (3), Feb (7), Mar (5), Apr (2), May (2), Jun (7), Jul (2), Aug (2), Sep (1), Oct (2)

#### Anax junius

(Drury, 1773)

7168C040-CAEB-57F8-8999-2AFA6FE7DDDD

##### Distribution

San Buenaventura, Jalisco, MX

##### Notes

Phenology in SBV: Aug (1)

#### Gynacantha helenga

Williamson & Williamson, 1930

612FF82A-49F3-52AD-891E-07C3FA1E4135

##### Distribution

San Buenaventura, Jalisco, MX

##### Notes

Phenology in SBV: Apr (1)

#### Remartinia luteipennis

(Burmeister, 1839)

AFC06648-741E-5996-84FC-BAA38FF51B51

##### Distribution

San Buenaventura, Jalisco, MX

##### Notes

Phenology in SBV: Jun (1), Sep (2)

#### Remartinia secreta

(Calvert, 1952)

5A3118E4-5ABB-5296-AC44-8DA54525B766

##### Distribution

San Buenaventura, Las Higueras, Jalisco, MX

##### Notes

Phenology in SBV: Jul (1), Aug (1), Sept (1)

#### Rhionaeschna multicolor

(Hagen, 1861)

916B6FD8-F8B8-58A6-9815-BD81390670F4

##### Distribution

San Buenaventura, Jalisco, MX

##### Notes

Phenology in SBV: May (1), Jun (1)

#### Rhionaeschna psilus

(Calvert, 1947)

56432FCC-B485-5AF1-AA92-5C12801B0DFF

##### Distribution

San Buenaventura, Amacuahutitlan, Jalisco, MX

##### Notes

Phenology in SBV: Feb (2), Sep (1)

#### Aphylla protracta

(Hagen in Sélys, 1859)

A2A67CF5-E661-5419-9E32-FD55BB641C13

##### Distribution

San Buenaventura, Jalisco, MX

##### Notes

Phenology in SBV: Aug (3)

#### Erpetogomphus elaps

Sélys, 1858

D1C87F1C-5CA9-59D8-9E83-BDE0696C742F

##### Distribution

San Buenaventura, Amacuahutitlan, Jalisco, MX

##### Notes

Phenology in SBV: Jul (1), Aug (1), Sep (9), Oct (2)

#### Phyllocycla elongata

(Sélys, 1858)

4D24F8D9-5A49-5BAD-9ACB-B182643DDE6B

##### Distribution

San Buenaventura, Amacuahutitlan, Jalisco, MX

##### Notes

Phenology in SBV: Aug (5), Sep (1), Oct (1)

#### Phyllogomphoides luisi

González y Novelo, 1990

B62D773A-0843-5D78-9B1E-9F39379B06F5

##### Distribution

San Buenaventura, Amacuahutitlan, Jalisco, MX

##### Notes

Phenology in SBV: Aug (1), Sep (5), Oct (1)

#### Phyllogomphoides pacificus

(Sélys, 1873)

6E99578B-8C6A-50FA-B067-C4EAA47F7025

##### Distribution

San Buenaventura, Amacuahutitlan, Las Higueras, Jalisco, MX

##### Notes

Phenology in SBV: Jun (1), Jul (3), Aug (26), Sep (10), Oct (11)

#### Progomphus belyshevi

Belle, 1991

3F01FB03-4902-5FC4-BE3D-19925060C7C9

##### Distribution

San Buenaventura, Jalisco, MX

##### Notes

Phenology in SBV: Aug (1)

#### Progomphus clendoni

Calvert, 1905

D81D524B-8B03-5AE1-B536-160E00093C0A

##### Distribution

San Buenaventura, Jalisco, MX

##### Notes

Phenology in SBV: Jun (1), Aug (1), Sep (3)

#### Brechmorhoga praecox

(Hagen, 1861)

229625DB-3250-5432-8E1D-A51BC4DC6076

##### Distribution

San Buenaventura, Amacuahutitlan, Jalisco, MX

##### Notes

Phenology in SBV: Feb (1), Aug (2)

#### Cannaphila insularis

Kirby,1889

277464BE-9F06-5CFE-BB28-D4D55364A92F

##### Distribution

Las Higueras, Jalisco, MX

##### Notes

Phenology in SBV: Aug (4), Sep (4)

#### Dythemis maya

Calvert, 1906

B266A5CB-A944-57E4-A01A-AF30D0F9E64D

##### Distribution

San Buenaventura, Amacuahutitlan, Jalisco, MX

##### Notes

Phenology in SBV: Aug (2), Sep (6), Oct (2)

#### Dythemis nigrescens

Calvert, 1899

60512A37-51BE-516F-B477-B8E3EFE52A4A

##### Distribution

San Buenaventura, Amacuahutitlan, Las Higueras, Jalisco, MX

##### Notes

Phenology in SBV: Dec (2), Jan (2), May (3), Jun (1), Jul (2), Aug (4), Sep (2), Oct (6)

#### Dythemis sterilis

Hagen, 1861

2D630FC4-FB98-5FC3-9F92-75E90AC9EC52

##### Distribution

San Buenaventura, Amacuahutitlan, Jalisco, MX

##### Notes

Phenology in SBV: Dec (6), Jan (2), Feb (1), Mar (1), Apr (2), Jun (2), Jul (2), Aug (2), Sep (2), Oct (1)

#### Erythemis haematogastra

(Burmeister, 1839)

B8329D39-2506-563F-8873-03FDF96C0171

##### Distribution

San Buenaventura, Jalisco, MX

##### Notes

Phenology in SBV: Dec (1)

#### Erythrodiplax basifusca

(Calvert, 1895)

941BF054-47C9-51D1-9DD0-D7BEA460016A

##### Distribution

San Buenaventura, Amacuahutitlan, Jalisco, MX

##### Notes

Phenology in SBV: Nov (1), Feb (3), Mar (10), Apr (6), May (2), Jun (8), Sep (1)

#### Erythrodiplax funerea

(Hagen, 1861)

65BACB76-BBD5-53AA-B58A-05B6E9F6B362

##### Distribution

San Buenaventura, Jalisco, MX

##### Notes

Phenology in SBV: Jun (2), Jul (1), Aug (3), Sep (1)

#### Libellula croceipennis

Sélys, 1868

52DBE0A4-8CF4-539D-9FDF-31090239AC7F

##### Distribution

San Buenaventura, Amacuahutitlan, Las Higueras, Jalisco, MX

##### Notes

Phenology in SBV: Jun (7), Aug (4), Sep (5), Oct (3)

#### Macrothemis hemichlora

(Burmeister, 1839)

F037D91A-D5B7-572B-BF3B-FB9A5BC532D4

##### Distribution

San Buenaventura, Jalisco, MX

##### Notes

Phenology in SBV: Feb (1)

#### Macrothemis inacuta

Calvert, 1898

C452BDB4-A91B-573B-9050-CF823ABE219F

##### Distribution

San Buenaventura, Jalisco, MX

##### Notes

Phenology in SBV: Nov (1), Dec (1), Feb (1), Jun (3), Jul (1), Aug (5), Sept (1)

#### Macrothemis inequiunguis

Calvert, 1895

2042CA7A-DD88-5FE9-AEED-9C9BABCD537E

##### Distribution

Amacuahutitlan, Jalisco, MX

##### Notes

Phenology in SBV: Jun (1)

#### Macrothemis pseudimitans

Calvert, 1898

9A803EC8-FCEE-5815-8520-C123EED1B939

##### Distribution

San Buenaventura, Amacuahutitlan, Jalisco, MX

##### Notes

Phenology in SBV: Nov (1), Dec (2), Mar (1), May (1), Jun (5), Jul (2), Aug (3), Sep (2)

#### Miathyria marcella

(Sélys in Sagra, 1857)

AFA02EFB-CCDC-5DE1-80EA-5E93067E0242

##### Distribution

San Buenaventura, Jalisco, MX

##### Notes

Phenology in SBV: Dec (4), Aug (1)

#### Micrathyria aequalis

(Hagen, 1861)

EF6038A9-B612-50AE-81BE-38A59A948307

##### Distribution

San Buenaventura, Jalisco, MX

##### Notes

Phenology in SBV: Dec (5), Aug (1), Sep (1), Oct (1)

#### Micrathyria didyma

(Sélys in Sagra, 1857)

3F5F8080-1376-5320-8FC1-C34423AD890F

##### Distribution

San Buenaventura, Jalisco, MX

##### Notes

Phenology in SBV: Jun (2), Jul (1), Aug (3), Sep (2)

#### Micrathyria paulsoni

González-Soriano, 2020

25729871-49C2-5070-BC2B-4E37AFA61542

##### Distribution

San Buenaventura, Jalisco, MX

##### Notes

Phenology in SBV: Jun (8), Jul (4), Aug (1), Sep (2)

#### Orthemis discolor

(Burmeister, 1839)

AC64915F-D6AA-55D9-B599-42700BE89A4C

##### Distribution

San Buenaventura, Amacuahutitlan, Las Higueras, Jalisco, MX

##### Notes

Phenology in SBV: Nov (1), Dec (4), Feb (3), Apr (2), Jun (3), Aug (2), Sep (1), Oct (8)

#### Orthemis ferruginea

(Fabricius, 1775)

7604C2A3-3365-5389-A8B6-FA98FD6BC6F3

##### Distribution

San Buenaventura, Amacuahutitlan, Jalisco, MX

##### Notes

Phenology in SBV: Nov (4), Dec (3), Jan (1), Feb 1), Jun (5), Jul (1), Sep (1)

#### Orthemis levis

Calvert, 1906

B0FD2362-5BB5-550A-955D-15C0272D6333

##### Distribution

San Buenaventura, Jalisco, MX

##### Notes

Phenology in SBV: Jun (14), Sept (1)

#### Pantala flavescens

(Fabricius, 1798)

63147C56-5E40-5CD5-A9B8-19D4E2CAC4E8

##### Distribution

San Buenaventura, Amacuahutitlan, Las Higueras, Jalisco, MX

##### Notes

Phenology in SBV: Aug (5)

#### Pantala hymenaea

(Say, 1840)

167C05C1-B21D-596A-AF55-96EFD9944E53

##### Distribution

San Buenaventura, Las Higueras, Jalisco, MX

##### Notes

Phenology in SBV: Aug (6)

#### Perithemis domitia

(Drury, 1773)

6FF663CC-589C-570C-A6E8-04969324950A

##### Distribution

San Buenaventura, Las Higueras, Jalisco, MX

##### Notes

Phenology in SBV: Jun (2), Aug (1)

#### Perithemis intensa

Kirby, 1889

736A74D7-169D-56AE-871A-B34A95EFDBF8

##### Distribution

San Buenaventura, Amacuahutitlan, Jalisco, MX

##### Notes

Phenology in SBV: Dec (5), Feb (3), Mar (2), Apr (3), May (2), Jun (3), Aug (1), Oct (5)

#### Perithemis tenera

(Say, 1840)

ED8F2AB2-F501-5988-9B53-33B8D614F8CC

##### Distribution

San Buenaventura, Jalisco, MX

##### Notes

Phenology in SBV: Feb (2)

#### Pseudoleon superbus

(Hagen, 1861)

4A708E15-0133-5CCF-940D-62CB7771C0BC

##### Distribution

San Buenaventura, Las Higueras, Jalisco, MX

##### Notes

Phenology in SBV: Nov (3), Dec (2), Mar (1), Aug (1)

#### Tauriphila azteca

Calvert, 1906

5AD9140E-6B59-5F0A-80F5-FA096972743E

##### Distribution

San Buenaventura, Jalisco, MX

##### Notes

Phenology in SBV: Jun (2), Aug (3)

#### Tramea onusta

Hagen, 1861

E2AF54A6-FBF1-5E2E-BE70-AC873969095C

##### Distribution

San Buenaventura, Jalisco, MX

##### Notes

Phenology in SBV: Aug (3), Sept (2)

## Analysis

### Species richness and diversity

**Figure 4.**
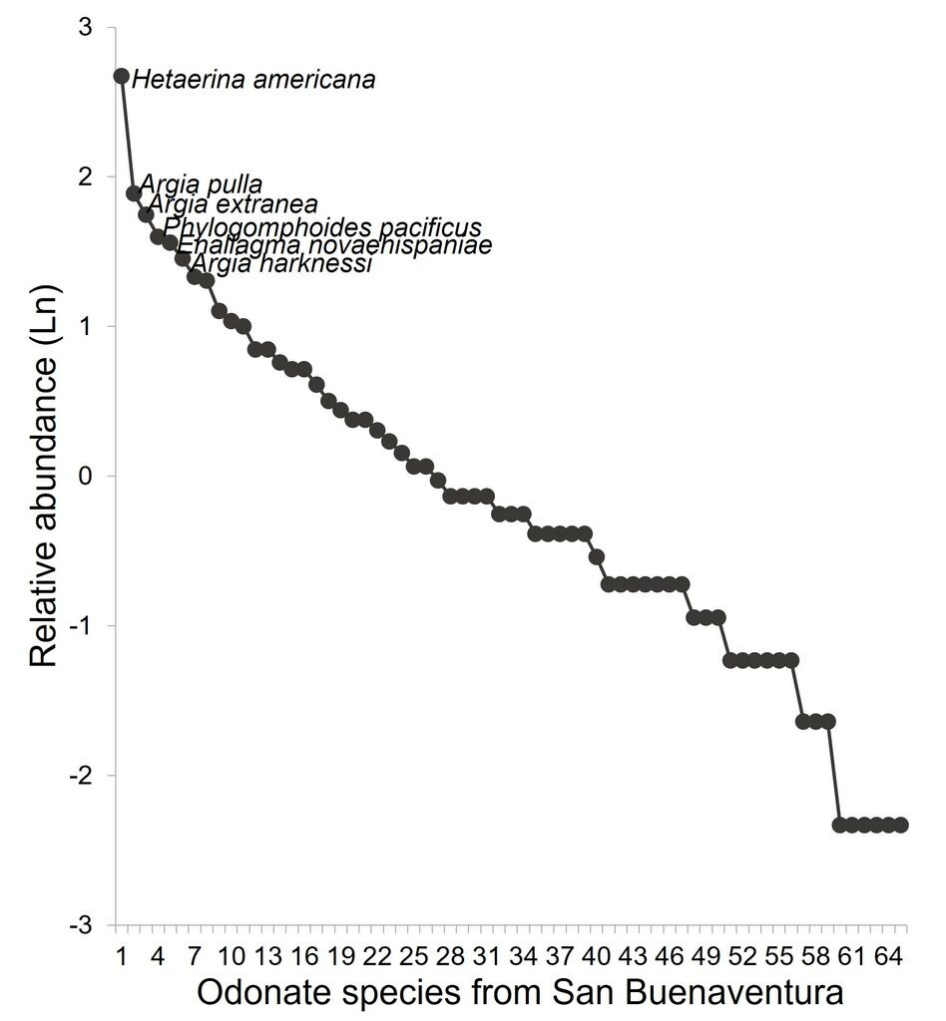
Species rank-abundance curve for the odonate species collected in San Buenaventura, Jalisco during 1996-1997.

**Figure 5.**
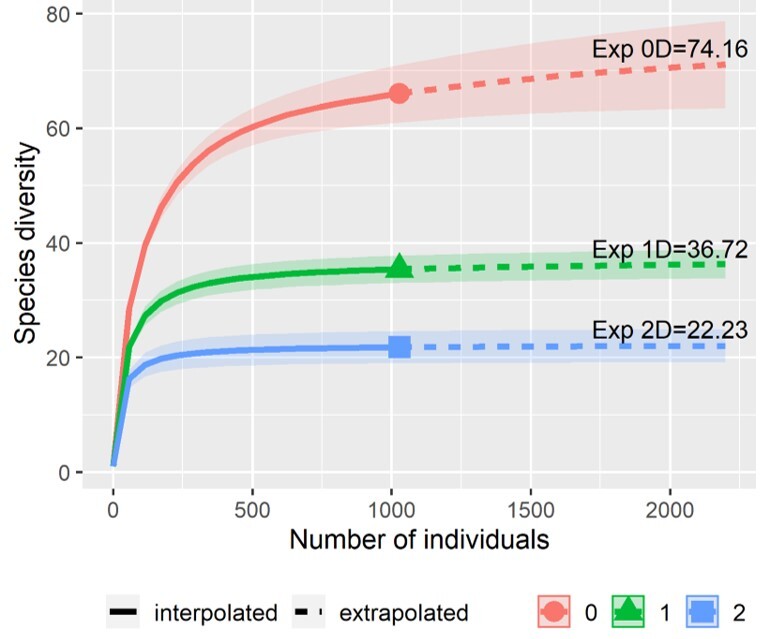
Interpolation-extrapolation cumulative curve, based on the number of odonate specimens collected in San Buenaventura, Jalisco (1996-1997) for three diversity orders: ^0^D, species richness; ^1^D, Shannon diversity; ^2^D, Simpson diversity. Exp, expected values of diversity of order 0, 1 and 2.

We documented a total of 1087 specimens belonging to seven families, 35 genera and 66 species of odonates in the assemblage (Figs 4, 5). Those values represent 87.5% of the families, 80% of the genera and 51% of the total species previously reported for the State of Jalisco (González-Soriano and Novelo-Gutiérrez 2014; Suppl. material 1). Libellulidae and Coenagrionidae were the families with the highest number of species, with 28 (42.4%) and 21 (31.8%), respectively, followed by Gomphidae (7), Aeshnidae (6), Calopterygidae (2), Lestidae (1) and Platystictidae (1). At the generic level, Libellulidae had the highest numbers of genera (15), followed by Coenagrionidae (8), Gomphidae (5), Aeshnidae (4), Lestidae (1), Calopterygidae (1) and Platystictidae (1). *Argia* was the most speciose genus with 10 species, followed by *Macrothemis* with four and the remaining genera with 1-3 species. *Phyllogomphoidesluisi* was recorded for the first time in the State of Jalisco and *Anisagrionallopterum* represented the first northernmost documented record of this species in America (Fig. 3). The total species richness (66 species) represented 88.9% from the total expected richness (^0^D, 74.16 effective species), 96.3% (35.4) of the expected Shannon diversity (^1^D) and 97.97% (22.2) of the expected Simpson diversity (^2^D) (Table 2, Fig. 5). The estimated species richness suggests that there could be another eight species in the site, whereas observed evenness (^1^D) and dominance (^2^D) showed values close to the estimated values of those diversity metrics.

**Figure 6.**
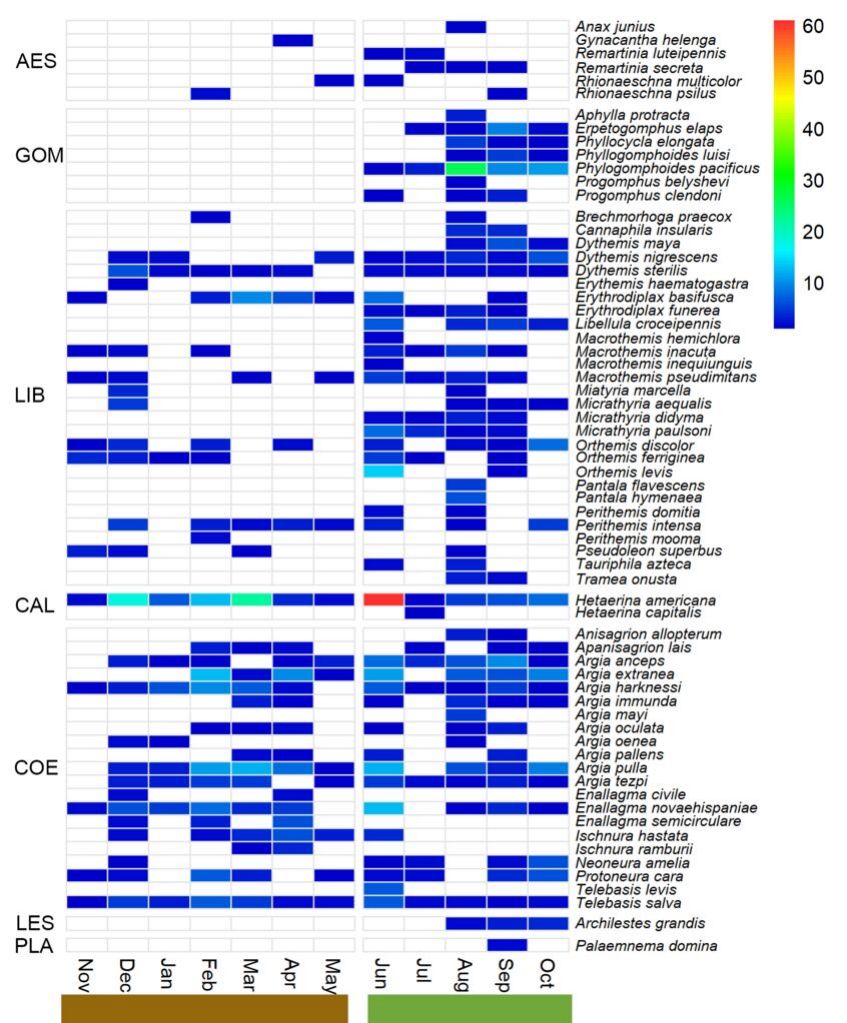
Heatmap showing monthly abundance (number of specimens) of odonate species collected in SBV, Jalisco. Species in rows are ordered according to their suborder and family. AES, Aeshnidae; GOM, Gomphidae; LIB, Libellulidae; CAL, Calopterygidae; COE, Coenagrionidae; LES, Lestidae; PLA, Platystictidae.

Species abundance during all the sampling was very heterogeneous. Only a few species were very abundant and most were represented by one or few specimens (Figs 4, 6). *Hetaerinaamericana* was by far the most abundant species with 149 specimens, followed by the coenagrionids *Argiapulla* (68), *A.extranea* (59), *Phyllogomphoidespacificus* (51), *Enallagmanovaehispaniae* (49) and *A.harknessi* (44). Those six species represented 41% of the total abundance of the assemblage (420 specimens). Surprisingly, one anisopteran, *P.pacificus*, appeared within the group of abundant species with more than 50 specimens. This contrasts with our other previous studies in TDF where the most abundant species belong to the suborder Zygoptera. On the contrary, 16 species were represented by only 1-3 specimens and contributed only 3% of the total abundance (31 specimens).

### Variation in temporal species and phylogenetic diversity

We observed a high variation in the different facets of diversity species of the odonate assemblage throughout the year. The highest species richness was recorded in August (44 species) and September (40), while the lowest was observed in November (11) and January (11). In addition, the highest value of abundance was observed in June (202 specimens), during the rainy season and the lowest in November (17) and May (21), during the dry season (Table 2, Fig. 6). The highest Shannon diversity was also documented in August and September, while the highest value of Simpson diversity was recorded in September (Table 2).

Phylogenetic divergence also showed a high variation throughout the year (Table 2): the taxonomic diversity showed its highest values from July to October; the taxonomic distinctness (Δ*) was higher in June and October. In general, it was high throughout the rainy period, whereas we observed lower values than the expected (76.42 species) from January to April except December. In general, the monthly taxonomic diversity (Δ) and the average taxonomic distinctness (Δ+) were lower than the expected.

### Relationship between odonate diversity and abiotic factors

Variation in precipitation showed a positive, moderate correlation with Shannon and Simpson diversities of the SBV odonate assemblage, as well as with phylogenetic divergence (Δ* and Δ+) (Table 3); whereas variation in minimum temperature also showed a positive, moderate correlation with monthly variation in species richness, Shannon diversity and all metrics of phylogenetic diversity (Table 3). Maximum temperature did not show any influence on the different metrics performed.

Monthly precipitation was strongly correlated with monthly minimum temperature (r = 0.833, P < 0.001); abundance showed a high, positive correlation with species richness and Shannon diversity (Table 3), which, in turn, were strongly correlated with Simpson diversity; also, taxonomic diversity and taxonomic distinctness showed a high, positive correlation between them. In addition, average taxonomic distinctness was highly and positively correlated with the diversity metrics of most species.

### Comparison with other TDF regions

In contrast with other TDF Mexican localities studied where coenagrionids were dominant (e.g. Dominguillo, Oaxaca; Huautla, Morelos; San Javier, Sonora), the odonate assemblage from SBV was dominated by one abundant calopterygid species: *Hetaerinaamericana* (Fig. 2). In addition, odonate species richness in SBV (66 species) was higher than the richness reported from Sierra de Huautla, Morelos (57) (González-Soriano et al. 2008); San Javier, Sonora (52) (González-Soriano et al. 2009); Río Pinolapa, Michoacán (51) (Novelo-Gutiérrez and Gómez-Anaya 2009); Dominguillo, Oaxaca (50) (González-Soriano et al. 2021); and Aguililla, Michoacán (40) (Novelo-Gutiérrez and Gómez-Anaya 2009). However, it was lower than that reported for Chamela, Jalisco (González-Soriano et al. 2004), which is the TDF site with the largest number of species recorded so far (78). SBV shares 71.2% species with Huautla; 63.6% with Chamela; 56.6% with Río Pinolapa; 53% with San Javier; 48.5% with Aguililla; and 42.4% with Dominguillo.

## Discussion

The high species richness found in SBV compared to other Mexican TDF assemblages (Chamela, Jalisco, which is the only locality with a higher species richness reported for Mexican TDF) seems to be explained by several factors. For instance, samplings were done along a greater diversity of aquatic habitats, including: (a) permanent ponds at the sides of the Ferreria river; (b) a permanent large river in SBV; (c) a shallow pond located along a narrow shady spring fed stream at Las Higueras, the habitat in which we found *Anisagrionallopterum*, their northernmost published record in Mexico (Fig. 3); and (d) a temporal stream in Los Yesos. Additionally, the presence of permanent semi-shaded ponds with abundant floating and rooted vegetation seems to influence the presence of more species of endophytic odonates, especially of the Aeshnidae family, with six species reported in SBV. Only in two of the previous studied localities (except for Chamela with 10 species and Huautla with 7 species), this family was as well represented as in SBV.

Temporal variation of abundance and species richness shows a pattern similar to other odonate and insect assemblages from the Mexican TDF, wherein higher values of richness and abundance were recorded during the rainy season (e.g. Odonata, González-Soriano et al. (2021)). However, those variables are not correlated with the variation in precipitation in SBV and only species richness is being influenced by minimum temperature. A different scenario can be seen in other diversity orders where the structure of the entire assemblage is analysed: evenness and dominance (^1^D and ^2^D in Table 3) do fluctuate with variation in precipitation and evenness also fluctuates with variation in monthly minimum temperature; i.e. higher levels of monthly precipitation lead to an increase in the monthly diversity and a more evenly distributed abundance of odonate species. In contrast, other odonate assemblages from the Mexican TDF forests show different phenological patterns than those from SBV, either showing an inverse pattern in which the highest values of species richness and diversity have been recorded at the end of the rainy season (e.g. Huautla, González-Soriano et al. (2008)) or showing a shorter period with high levels of richness, abundance and diversity (e.g. Dominguillo, González-Soriano et al. (2021)).

In addition, variation in precipitation and minimum temperatures also influence the variation in the taxonomic structure of the SBV odonate assemblage: the higher the precipitation, the greater the taxonomic distance amongst odonate specimens and the more evenly distributed the abundances are amongst odonate species in the taxonomic hierarchy of the whole assemblage. Additionally, higher values of minimum temperature lead to greater taxonomic distances amongst odonate species within the assemblage structure. A similar pattern has been previously recorded for Santiago Dominguillo, Oaxaca and it is likely associated to a higher availability of niches and resources during the rainy season than that of the dry season (González-Soriano et al. 2021).

On the other hand, we found that minimum temperature was more informative than the maximum temperature values recorded in the sampling year, which suggest that it might be more convenient for odonate and other insect assemblages to explore other climatic variables associated with their diversity patterns (e.g. average monthly temperature) as those variables could be more biologically meaningful. In addition, some diversity metrics were redundant amongst them: it seems that the most informative and non-redundant metrics for the SBV odonate assemblage were Shannon diversity and taxonomic diversity metrics. Choosing the metrics that are the most complementary and informative will help us achieve a better understanding of the structure of ecological communities and the factors influencing them.

In SBV, some odonate families (such as Gomphidae, Lestidae and Platystictidae) exhibited a more seasonal pattern than the others and were recorded only during the rainy season. Gomphidae and Platystictidae have also been mainly recorded during that season in other TDF assemblages, such as San Javier (Sonora), Chamela (Jalisco) and Dominguillo (Oaxaca) (González-Soriano et al. 2004, González-Soriano et al. 2009, González-Soriano et al. 2021). Conversely, many Coenagrionidae species can be found throughout most of the year.

## Supplementary Material

XML Treatment for Archilestes grandis

XML Treatment for Palaemnema domina

XML Treatment for Hetaerina americana

XML Treatment for Hetaerina capitalis

XML Treatment for Anisagrion allopterum

XML Treatment for Apanisagrion lais

XML Treatment for Argia anceps

XML Treatment for Argia carlcooki

XML Treatment for Argia extranea

XML Treatment for Argia harknessi

XML Treatment for Argia mayi

XML Treatment for Argia oculata

XML Treatment for Argia oenea

XML Treatment for Argia pallens

XML Treatment for Argia pulla

XML Treatment for Argia tezpi

XML Treatment for Enallagma civile

XML Treatment for Enallagma novaehispaniae

XML Treatment for Enallagma semicirculare

XML Treatment for Ischnura hastata

XML Treatment for Ischnura ramburii

XML Treatment for Neoneura amelia

XML Treatment for Protoneura cara

XML Treatment for Telebasis levis

XML Treatment for Telebasis salva

XML Treatment for Anax junius

XML Treatment for Gynacantha helenga

XML Treatment for Remartinia luteipennis

XML Treatment for Remartinia secreta

XML Treatment for Rhionaeschna multicolor

XML Treatment for Rhionaeschna psilus

XML Treatment for Aphylla protracta

XML Treatment for Erpetogomphus elaps

XML Treatment for Phyllocycla elongata

XML Treatment for Phyllogomphoides luisi

XML Treatment for Phyllogomphoides pacificus

XML Treatment for Progomphus belyshevi

XML Treatment for Progomphus clendoni

XML Treatment for Brechmorhoga praecox

XML Treatment for Cannaphila insularis

XML Treatment for Dythemis maya

XML Treatment for Dythemis nigrescens

XML Treatment for Dythemis sterilis

XML Treatment for Erythemis haematogastra

XML Treatment for Erythrodiplax basifusca

XML Treatment for Erythrodiplax funerea

XML Treatment for Libellula croceipennis

XML Treatment for Macrothemis hemichlora

XML Treatment for Macrothemis inacuta

XML Treatment for Macrothemis inequiunguis

XML Treatment for Macrothemis pseudimitans

XML Treatment for Miathyria marcella

XML Treatment for Micrathyria aequalis

XML Treatment for Micrathyria didyma

XML Treatment for Micrathyria paulsoni

XML Treatment for Orthemis discolor

XML Treatment for Orthemis ferruginea

XML Treatment for Orthemis levis

XML Treatment for Pantala flavescens

XML Treatment for Pantala hymenaea

XML Treatment for Perithemis domitia

XML Treatment for Perithemis intensa

XML Treatment for Perithemis tenera

XML Treatment for Pseudoleon superbus

XML Treatment for Tauriphila azteca

XML Treatment for Tramea onusta

5FE6C06D-02B4-5CA2-BA00-8C39DF17E26A 10.3897/BDJ.12.e116135.suppl1 Supplementary material 1 Species richness by family from the State of Jalisco and San Buenaventura locality Data type Table Brief description In parentheses, the proportion of SBV species in relation to Jalisco diversity, based on González-Soriano & Novelo-Gutierrez (2013) and González-Soriano, unpublished data. File: oo_935718.docx https://binary.pensoft.net/file/935718 González-Soriano, E

## Figures and Tables

**Table 1.** San Buenaventura, Jalisco localities where odonate sampling collection was performed (1996-1997).

**Sampling locality**	**Municipality**	**Coordinates**
San Buenaventura, town	El Limón	19.79357°N, -104.0554°W
Los Yesos, El Limón	El Limón	19.7511°N, -104.0592°W
Las Higueras, Ejidal pools	El Limón	19.80578°N, -104.02077°W
Amacuahutitlán	Tonaya	19.81593°N, -104.1374°W

**Table 2.** Monthly values of temperature (°C) and precipitation (mm), odonate species diversity and phylogenetic diversity from SBV during 1996-1997. Tmax, mean maximum temperature; Tmin, mean minimum temperature; PPM, mean precipitation; N, abundance (specimen count); ^0^D, species richness; ^1^D, Shannon diversity; ^2^D, Simpson diversity; Δ, taxonomic diversity; Δ*, taxonomic distinctness; Δ+, average taxonomic distinctness. Additionally, expected values of different measures of diversity correspond to the whole assemblage (^0^D, ^1^D, ^2^D) or by month (Δ, Δ*, Δ+).

**Month**	**Tmax**	**Tmin**	**PPM**	**N**	**^0^D**	**^1^D**	**^2^D**	**Δ**	Δ*	**Δ**+
Nov	31.76	13.64	13.5	17	11	9.44	8.1	73.44	79.13	79.42
Dec	31.32	12.39	0	83	24	18.40	13.21	75.71	80.97	76.08
Jan	29.68	9.85	2	31	11	9.03	7.81	63.71	70.92	70.39
Feb	32.64	11.63	0	94	22	15.6	12.61	63.68	68.48	76.26
Mar	33.45	14.82	30.5	82	19	11.99	8.46	65.70	73.66	69.67
Apr	32.3	15.03	52	68	20	15.83	13.24	63.32	67.51	69.71
May	37	17.1	58	21	13	11.76	10.79	75.51	79.60	78.24
Jun	34.7	20.64	178.5	202	36	18.33	9.52	73.50	81.75	77.36
Jul	32.13	18.76	256.5	35	21	18.58	16.49	78.03	80.82	81.62
Aug	32.97	18.86	92.5	137	44	29.24	17.91	76.16	80.10	78.37
Sep	32.4	19.57	240	119	40	30.35	24.32	78.14	80.82	80.21
Oct	31.35	16.5	69	89	24	17.03	14.01	77.67	82.72	80.06
Expected values					74.16	36.72	22.23	81.58	76.42	80.37

**Table 3.** Pearson correlation coefficients between temperature, precipitation and odonate species diversity and phylogenetic diversity from SBV and amongst diversity metrics performed. Tmax, mean maximum temperature; Tmin, mean minimum temperature; PPM, mean precipitation; N, abundance; ^0^D, species richness; ^1^D, Shannon diversity; ^2^D, Simpson diversity; Δ, taxonomic diversity; Δ*, taxonomic distinctness; Δ+, average taxonomic distinctness. Additionally, expected values of different measures of diversity correspond to the whole assemblage (^0^D, ^1^D, ^2^D) or by month (Δ, Δ*, Δ+). ^⁎^P < 0.05, ^⁎⁎^P < 0.01.

	**Abiotic factors**			**Species diversity**				**Phylogenetic diversity**	
	**Tmax**	**Tmin**	**PPM**	**N**	**^0^D**	**^1^D**	**^2^D**	**Δ**	Δ*
**N**	0.198	0.531	0.348						
**^0^D**	0.127	0.677*	0.551	0.847**					
**^1^D**	0.032	0.636*	0.609*	0.612*	0.931**				
**^2^D**	-0.055	0.529	0.644*	0.289	0.701**	0.903**			
**Δ**	0.196	0.659*	0.580*	0.148	0.431	0.526	0.529		
**Δ** *	0.209	0.629*	0.501	0.243	0.34	0.401	0.321**	0.954**	
**Δ** +	0.126	0.699*	0.587*	0.821**	0.996**	0.942**	0.729**	0.493	0.454
