# Supplementary material for: Diversity of an Odonata assemblage from a tropical dry forest in San Buenaventura, Jalisco, Mexico (Insecta, Odonata)
Source: Biodivers Data J. 2024 Feb 23;12:e116135. doi: 10.3897/BDJ.12.e116135 (PMC10907955; doi:10.3897/BDJ.12.e116135)
Supplement: Supplementary material 1 — Species richness by family from the State of Jalisco and San Buenaventura locality [file bdj-12-e116135-s001.docx]

**González-Soriano et al. An Odonata Assemblage from a Tropical Dry Forest in San Buenaventura, Jalisco, Mexico (Insecta: Odonata)**

**Supplementary material.**

**Table S1. Species richness by family from the state of Jalisco and San Buenaventura Jalisco.** In parentheses, the proportion of SBV species in relation to Jalisco diversity based on González-Soriano & Novelo-Gutierrez (2013) and González-Soriano, unpublished data.

| **Families** | **San Buenaventura** | **Jalisco** |  |
| --- | --- | --- | --- |
| Lestidae | 1(20%) | 5 |  |
| Calopterygidae | 2(28.5%) | 7 |  |
| Coenagrionidae | 21(49%) | 43 |  |
| Platystictidae | 1 (100%) | 1 |  |
| Aeshnidae | 6 (37.5) | 16 |  |
| Gomphidae | 7(44%) | 16 |  |
| Libellulidae | 28 (45%) | 62 |  |
| Total | 66 | 150 |  |
